# Quantifying household liquid medicine dosing errors in Sri Lanka: A hidden public health risk

**DOI:** 10.1371/journal.pgph.0005841

**Published:** 2026-02-06

**Authors:** Manori Jayasinghe, Hasiduni Madhushika, Kanchana Wijesekera, Sewwandi Subasinghe

**Affiliations:** Department of Pharmacy, Faculty of Allied Health Sciences, University of Ruhuna, Galle, Sri Lanka; McGill University, CANADA

## Abstract

Inaccurate measurement of liquid medicines remains a critical yet underrecognized patient safety concern in low-resource settings. In Sri Lanka, despite the adoption of metric units in formal healthcare, the use of uncalibrated household items such as teaspoons and tablespoons remains widespread. Bulk dispensing without standardized measuring aids is common, and dosing instructions often rely on informal household measures. This study assessed the volume accuracy of commonly used household liquid medicine measuring tools in a Sri Lankan suburb, highlighting their potential contribution to dosing errors. A cross-sectional survey of 50 households in Karapitiya, Galle District, Sri Lanka, was conducted. Frequently used measuring devices were tested using validated weighing and volumetric methods. Accuracy was evaluated against United States Pharmacopoeial (USP) specifications for acceptable deviation limits. Only 53.4% of measuring cups met USP accuracy standards across all tested volumes, although 86.2% complied for the 5.00 mL mark. Meanwhile, 62.5% of graduated measuring spoons failed to meet the standard. Household teaspoons and tablespoons demonstrated substantial variability, with volumes ranging from 2.893–7.759 mL and 4.252–15.043 mL, respectively. Over 93% of both spoon types delivered less than their expected standard volumes. The study discloses widespread inaccuracy in common dosing tools used in Sri Lankan households, posing avoidable risks to medication safety. It calls for urgent regulatory action to mandate standardized, calibrated devices and integrate user education into prescribing and dispensing practices. These findings point out a critical safety gap in primary care, particularly in low- and middle-income settings.

## Introduction

Accurate dosing of oral liquid medicines remains a critical yet underexamined challenge, particularly for children, due to inconsistencies in measuring devices and user practices. This variability presents a preventable but persistent threat to patient safety. The accurate delivery of oral liquid drugs presents a more intricate challenge compared to other dosage forms. Inaccuracies in volumetric measurements and dosing devices are key contributors to incorrect dosing in children [1]. Both user proficiency and device accuracy are crucial in delivering the correct dose. To enhance dosing accuracy, oral liquid medications are often supplied with devices designed for precise measurement. However, in many low-resource settings, such devices are often absent, inadequate, or not used as intended, leaving caregivers to rely on informal household tools [2–4]. The present cross-sectional study investigates the measurement accuracy of liquid dosing aids commonly used in Sri Lankan households.

Despite official adoption of metric units in formal healthcare policy, the use of household items (e.g., teaspoons, tablespoons) persists in both prescribing and dispensing practices, as well as in community and home-based medicine use [1,5,6]. Yet, few empirical studies have systematically evaluated the actual measurement accuracy of these tools in real-world household settings, particularly in Sri Lanka. Higher usage of household spoons has been observed in studies conducted in Japan, the UK, and elsewhere [7–9]. Recent literature highlights that not all medicines are supplied with dosing devices. For example, Saaka et al. (2022) reported that 78.6% of oral medicines in Ghana lacked measuring aids, while 20% of medications in Sri Lanka were supplied without such devices [2,3]. A study in New Jersey found that only 12.8% of products included measuring devices [4]. Johnson et al. (2016) emphasized that patients often turn to household spoons due to inconsistent packaging, leading to variable administration practices. Furthermore, manufacturers often overlook the crucial aspect that a dosing device should be used exclusively with the product it is supplied with, justifying caregivers’ and patients’ inclination to utilize these devices with other liquid medicines [3].

Johnson et al. (2016) highlighted bulk dispensing practices, where multiple prescriptions are filled from one container. Even if a dosing aid is supplied, patients may not receive it if the product is repacked. Additionally, professional advice can contribute to this problem; Wojewoda et al., 2017 reported that 35.3% of pharmacists in Bronx, New York, sometimes advised the use of a household spoon to measure liquid medicines [10]. Another issue lies in the inaccuracies of the dosing devices supplied. The FDA has acknowledged that many liquid medicines are marketed with devices incompatible with product dosage instructions [6]. This problem is particularly prevalent in low- and middle-income countries. For instance, a study in Ghana found significant discrepancies in the volumes measured by cups and spoons compared to the intended 5 mL dose [3]. Similar findings have shown the risk of overdosing when using measuring cups for pediatric medications [3].

In Sri Lanka, both household spoons and graduated measuring devices are used. As the country provides free healthcare, liquid medicines are often dispensed from bulk containers without dosing devices, and labels frequently indicate doses in teaspoons or tablespoons. Additionally, the Sri Lankan pharmaceutical market sources products globally, increasing variability in dosing devices. The heterogeneity in sourcing, coupled with bulk dispensing practices, further undermines the standardization of dosing aids, revealing a systemic gap in regulatory and pharmacy-level oversight. However, few experimental studies have systematically evaluated the real-world accuracy of commonly used measuring devices in Sri Lankan households.

This study evaluates the accuracy of both product-supplied and household liquid dosing devices used by caregivers in Sri Lanka, applying experimental methods to produce localized, device-level evidence on a widely acknowledged but insufficiently addressed risk. The findings aim to catalyze policy and regulatory attention toward ensuring calibrated dosing aids are supplied with all liquid medications, and to raise awareness about the avoidable risks embedded in everyday dosing practices. This study intends to facilitate communication among stakeholders involved in treatment, production, regulation, and control contributing to the minimization of pharmaceutical errors brought on by overlooked yet vital measuring devices.

## Materials and methods

**Fig 1:**
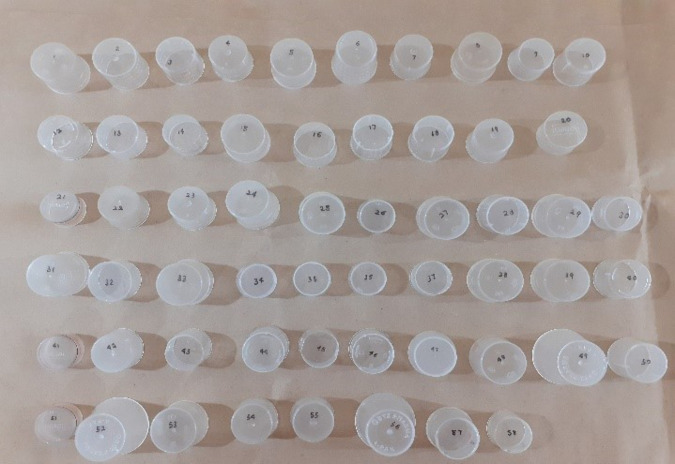
Graduated measuring cups.

**Fig 2:**
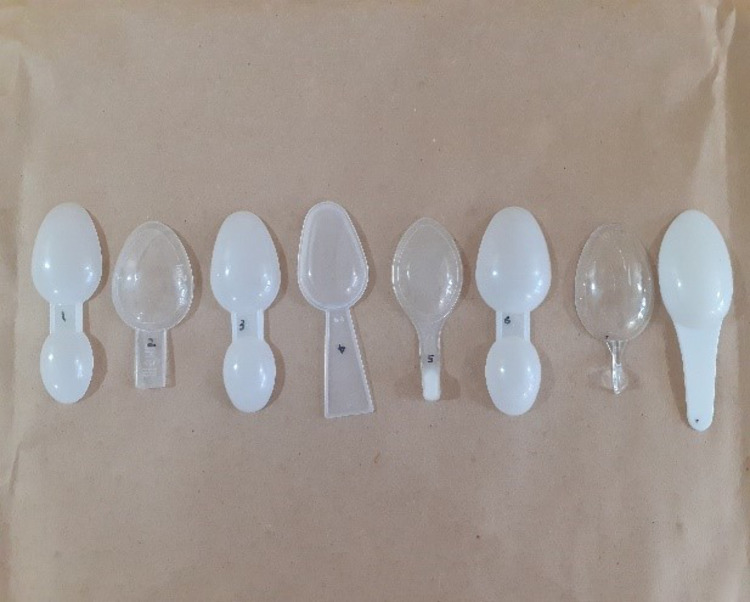
Graduated measuring spoons.

**Fig 3:**
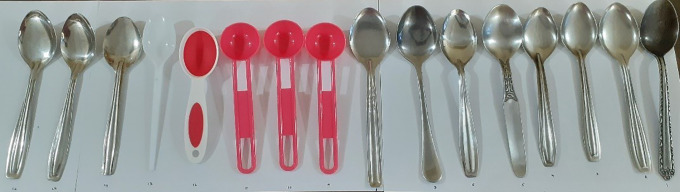
Household tablespoons.

**Fig 4:**
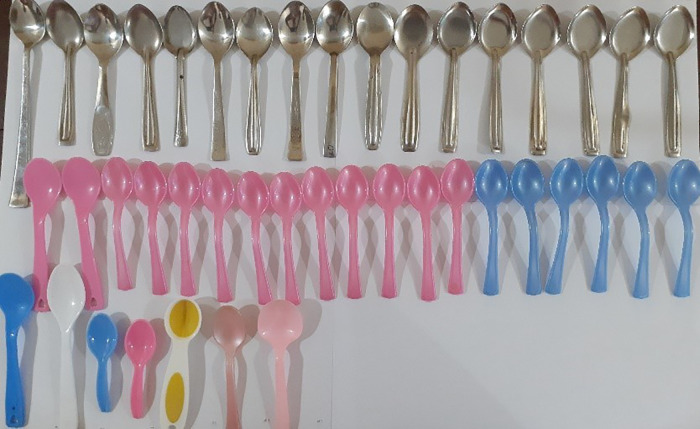
Household teaspoons.

This cross-sectional study with experimental validation of device accuracy was conducted in Karapitiya, a suburban community in Galle District, Sri Lanka. Fifty households were recruited through convenience sampling via community-based outreach in the local area by a trained pharmacy graduate researcher (HM). Households were eligible for inclusion if they: (1) had used liquid medications within the previous six months, (2) possessed measuring devices (cups, spoons, or other tools) used for medication administration, and (3) either possessed alternative measuring devices or no longer required the device for medication use. After explaining study objectives and obtaining informed consent, measuring devices from each household were collected, a unique identification number was allocated (Figs 1–4) and brought to the laboratory for accuracy testing. This sampling approach prioritized obtaining devices actually in use in routine community practice, providing ecologically valid data on real-world measurement tool accuracy.

This study did not focus on collecting product-specific measuring devices (i.e., dosing devices supplied with specific branded liquid medications) for several reasons reflecting Sri Lankan dispensing practices. First, bulk dispensing is standard practice in Sri Lanka’s free healthcare system, where liquid medicines are dispensed from large containers and repackaged into smaller quantities. Product-specific measuring devices are typically not transferred to patients during this repackaging process. Second, even when devices are initially supplied with commercial products, they are often separated from the medication during storage or use, and households tend to reuse the same measuring device for multiple different liquid medications.

Of the 50 households visited, product-specific measuring devices (such as dosing syringes or cups supplied with commercial products) were found in approximately 5 households (10%). However, participants reported that these devices were either not regularly used or were used interchangeably with household measuring tools rather than exclusively with their original product. Given this practice pattern, the study focused on the devices actually used for medication administration in routine practice; graduated cups, graduated spoons, and household teaspoons and tablespoons, regardless of their origin.

To enhance measurement accuracy, an evaluation of each measuring device was conducted using two methods: the weighing method and the volumetric method. Each measurement was done in triplicate, averaged, and conducted independently by two investigators (HM and MJ), to ensure accuracy and reproducibility. Yellow-colored distilled water was used to enhance visual contrast, and all measurements were performed under controlled laboratory conditions in the research lab at the Faculty of Allied Health Sciences, University of Ruhuna, maintained at room temperature (25°C) to ensure consistency and accuracy across trials.

### Weighing Method

For the accuracy assessment of graduated measuring cups (n = 58) and spoons (n = 8), the initial weights of the empty dosing devices were determined using an analytical balance validated according to the single-trial validation method. The devices were then filled with purified water at calibration marks indicating minimum, 5 mL, and maximum volumes. Each measurement was repeated three times, and between each measurement, the devices were thoroughly wiped to eliminate any traces of water from the previous measurement. The weight difference (denoted as ‘m’) between empty and filled devices was calculated, and the volume (v) was determined using the equation v = m/ρ, where ‘ρ’ is the density of water (1.0 g/mL at 25°C).

For teaspoons (n = 45) and tablespoons (n = 15), a standard container was used, with its initial mass measured using a balance validated by the single-trial method. After being filled with a spoonful of water, the container was reweighed. Each measurement was repeated three times and averaged. The volume (v) was determined using the same equation. Only fully filled spoon volumes were analyzed due to household spoons’ inability to measure smaller amounts.

## Volumetric method

For the accuracy assessment of graduated measuring cups (n = 58) and spoons (n = 8) using the volumetric method, the cups were filled with colored water up to minimum, 5 mL, and maximum marks. The liquid was then transferred into a standard graduated cylinder, and measurements were recorded in three trials.

For teaspoons (n = 45) and tablespoons (n = 15), they were filled to their maximum volume with colored water, and the water was carefully poured into a standard graduated cylinder. Measurements were recorded and repeated three times. Only fully filled spoon volumes were considered, with standard volumes of 5.0 mL and 15.0 mL.

### Methodological accuracy of two test methods

**Fig 5:**
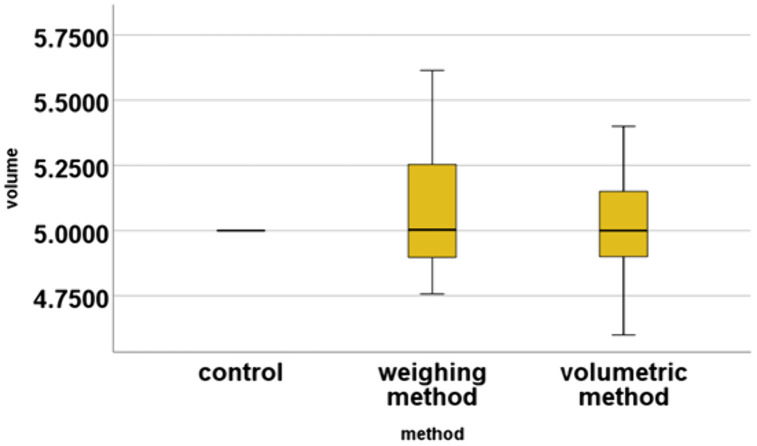
Comparison of volumes measured by each testing method.

Before the main test, the methodological accuracy of the two test methods was confirmed by assessing 10% of the samples (n = 7) for 5 mL volume accuracy using both the volumetric and weighing methods. A one-way ANOVA test was conducted to compare the measured 5 mL volume from both methods with the expected volume [11]. The results indicated no statistically significant difference between the volumes measured by the two methods and the expected volume (p = 0.735). (Fig 5).

Subsequently, the consistency between the volumetric method and weighing methods was further assessed using paired-samples t-test. The test results indicated no significant difference between the two methods (p = 0.759), confirming that both methods provide comparable volume measurements.

### Calculation of the deviation of the measured volume from the expected volume

The United States Pharmacopoeial (USP) specifications were used to calculate the deviation between measured and expected volumes. According to the USP, volume errors for liquid measurements using graduated devices should not exceed 10% of the indicated amount under typical use conditions. Devices with deviations over 10% failed the accuracy test. The following equation provided by the USP was used to calculate the percentage deviation [12].


$$\%\;\text{Deviation}\;=\;\frac{\text{Measured}\;\text{Volume}\;-\;\text{Expected}\;\text{Volume}}{\text{Expected}\;\text{Volume}}\times \text{1}\text{0}\text{0}$$

## Results

This study examined the liquid measurement accuracy of graduated measuring cups (n = 58), graduated measuring spoons (n = 8), teaspoons (n = 45), and tablespoons (n = 15) used for liquid medicine dosing using the weighing method and volumetric method.

### Accuracy of minimum volumes measured by measuring cups

This subsection evaluated the smallest marked volume on each measuring cup to determine its accuracy at the lower end of the dosing range.

Of the 58 measuring cups that have been used in the study, 31.0% were recorded as having a volume less than the expected minimum volume of particular measuring cups, and 68.9% higher than the expected minimum volume. Measuring cups with their minimum volume indicated as 2.0 mL (n = 7) showed the mean ± SD volume of 2.192 ± 0.63 mL, 2.5 mL cups (n = 46) showed 2.670 ± 0.30 mL, and 5.0 mL (n = 5) minimum volume cups showed 5.513 ± 0.19 mL.

**Fig 6:**
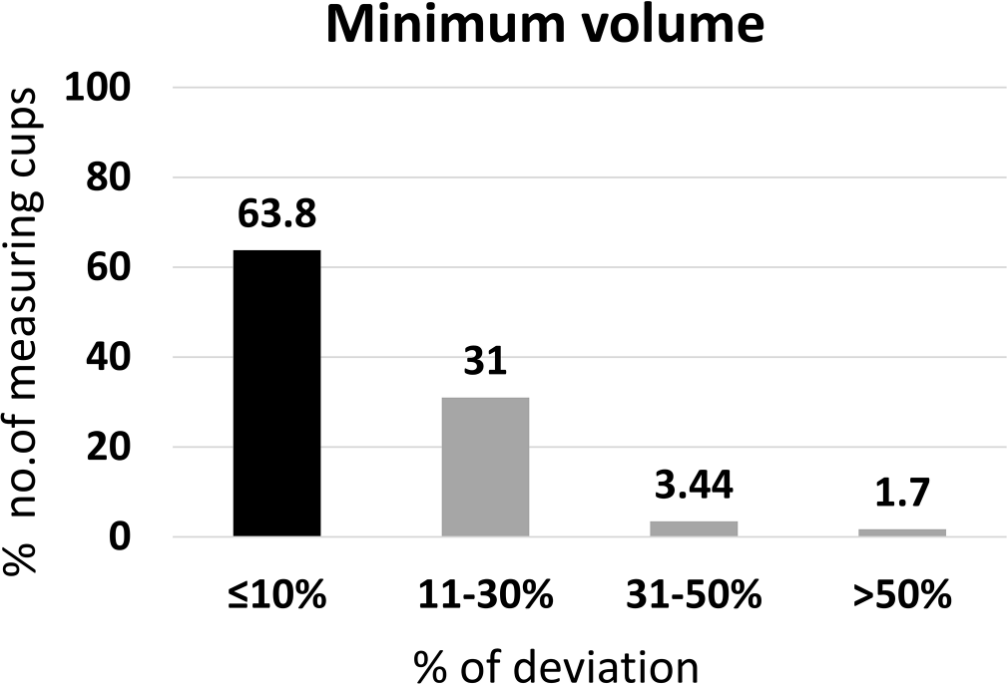
Accuracy of the minimum volume of household measuring devices (n = 58).

From the 58 measuring cups, 63.8% had a ≤ 10% deviation from the expected minimum volume of each measuring cup and passed the USP criteria. Further, 31.0% were in the 11–30% deviation, 3.44% were in the 31–50% deviation, and 1.70% were in the ≥ 50% (Fig 6).

These results indicate that while most cups performed adequately at low volumes, a significant proportion exceeded the acceptable deviation limits.

### Accuracy of 5 mL volumes

The accuracy of the 5 mL mark was evaluated as a commonly used dosing volume in clinical and household settings.

From the 58 measuring cups that have been used in the study, 36.2% recorded as volume less than the 5 mL volume of particular measuring cups, 1.72% recorded as volume same as the 5.0 mL, and 65.5% higher than the 5.0 mL volume. The mean ± SD of the volumes measured by 5 mL measuring cups was 5.059 ± 0.53 mL.

**Fig 7:**
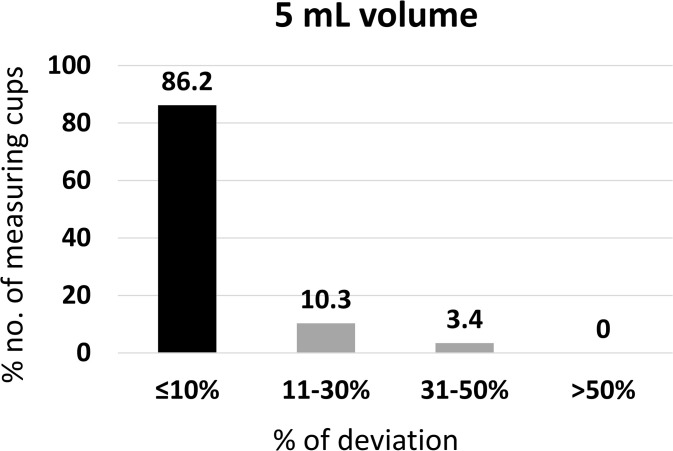
Accuracy of 5 ml volume of household measuring devices (n = 58).

Of the 58 measuring cups, 86.2% contained ≤10% deviation from the expected 5 mL volume of each measuring cup and passed the USP criteria. Further, 10.3% were within the range of 11–30% deviation. Two cups (3.4%) were in the 31–50% deviation and none of the measuring cups deviated >50% from the expected volume (Fig 7).

These findings highlight the relatively higher accuracy of cups at this standard dosing volume compared to the minimum mark.

### Accuracy of maximum volumes

This subsection assesses whether measuring cups maintained accuracy at their upper volume limits, which is important when dosing larger quantities.

Of the 58 measuring cups, 65.5% recorded a volume less than the expected maximum volume of particular measuring cups and 34.5% (n = 20) higher than the expected maximum volume. Measuring cups with their maximum volume indicated as 5.0 mL (n = 4) showed the mean ± SD measuring volume of 5.162 ± 0.12 mL, 10.0 mL cups (n = 42) showed 9.756 ± 0.63 mL, 15.0 mL cups (n = 9) showed 14.832 ± 0.63 mL and 30.0 mL maximum volume cups (n = 3) showed 29.871 ± 0.32 mL.

**Fig 8:**
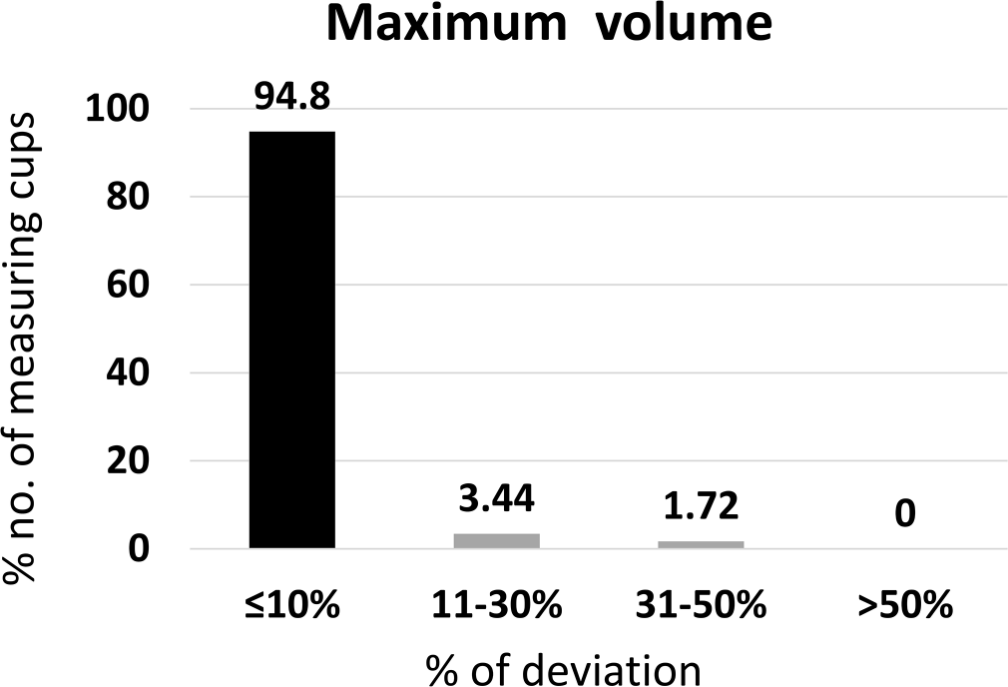
Accuracy of the maximum volume of household measuring devices (n = 58).

Of the 58 measuring cups, 94.8% contained ≤10% deviation from the expected maximum volume of each measuring cup and passed the USP criteria. Further, 3.44% were in the 11–30% deviation, 1.72% were in the 31–50% deviation, and none of the measuring cups deviated >50% (Fig 8).

The results suggest that accuracy at the maximum volume was the most consistent among the three volume categories tested.

### Accuracy of measuring spoons

#### Accuracy of 2.5 mL volumes.

This subsection evaluated whether measuring spoons marked for 2.5 mL delivered volumes were within USP-accepted limits.

From the measuring spoons (n = 8) that were used in the study, 75% were less than the expected volume (2.5 mL), and 25% were higher than the expected volume. The mean ± SD of the measurement taken for the 2.5 mL volume was 2.360 ± 0.41 mL.

**Fig 9:**
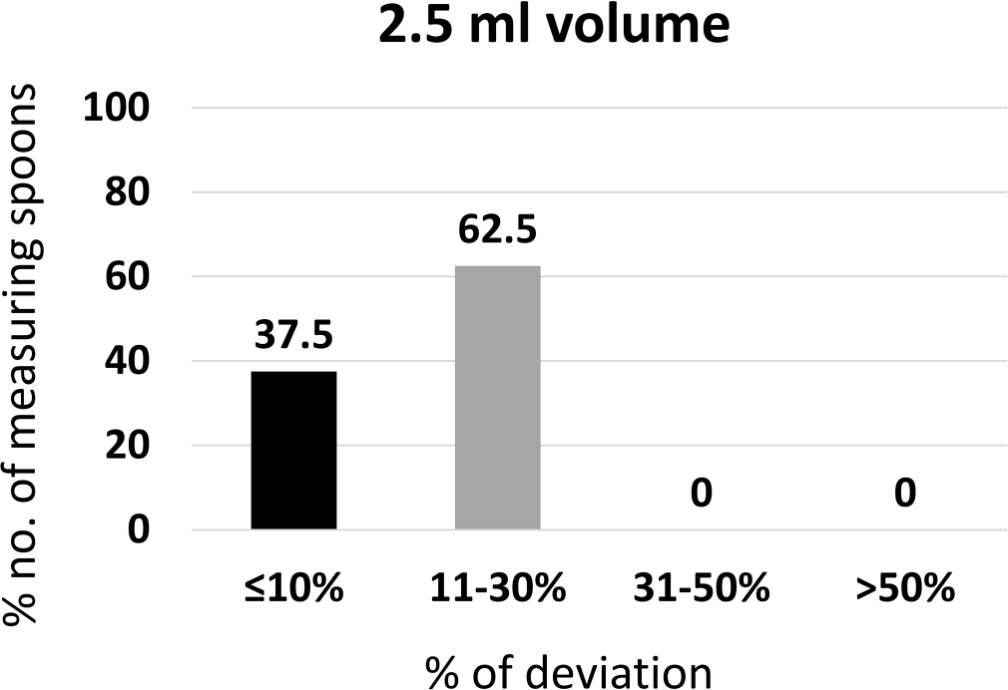
Percentage of measuring spoons meeting USP accuracy criteria at 2.5 ml volume (n = 8).

Of the 8 measuring spoons, 37.5% had a ≤ 10% deviation from their expected volume and complied with the USP criteria for measuring spoons. However, 62.5% were within the deviation of 11–30% (Fig 9).

These results suggest that the majority of 2.5 mL spoons underperformed in terms of accuracy, failing to meet USP standards.

#### Accuracy of 5.0 mL volumes.

The 5.0 mL volume, representing a standard teaspoon dose, was tested for accuracy using the same set of graduated measuring spoons.

Among the eight graduated measuring spoons tested for 5.0 mL volume accuracy, 75.0% were recorded as less than the expected volume (5.0 mL), and 25.0% were higher than the expected 5.0 mL. The mean ± SD of the measurement taken for the 5.0 mL volume was 5.017 ± 0.44 mL.

**Fig 10:**
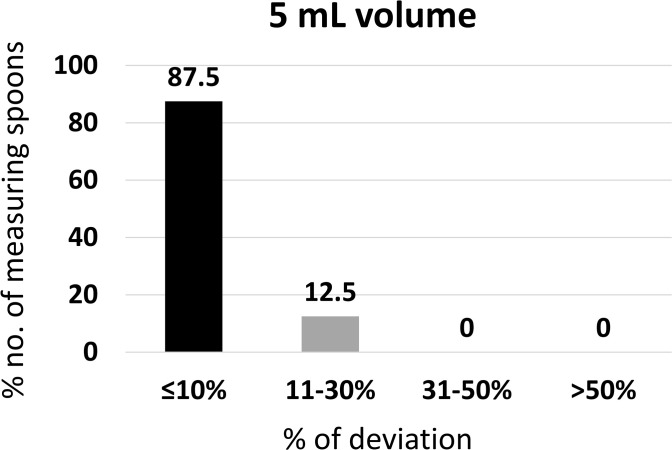
Percentage of measuring spoons meeting USP accuracy criteria at 5 ml volume (n = 8).

Further, of the 8 measuring spoons, 87.5% had a ≤ 10% deviation from their expected volume and complied with the USP criteria for measuring spoons. However, 12.5% were within the deviation of 11–30% (Fig 10).

These findings indicate that 5.0 mL spoons demonstrated acceptable accuracy overall, with most falling within USP-defined limits.

#### Accuracy of tablespoons.

This subsection evaluated the accuracy of household tablespoons, using 15 samples tested by the weighing method.

The volume of tablespoons measured using the weighing method ranged between 4.25 mL to 15.04 mL. From the 15 tablespoons that were used in the study, 93.3% were recorded as having a volume less than the expected 15.00 mL volume and 6.67% was higher than the volume. Tablespoons showed the overall mean ± SD value of 9.00 ± 3.39 (n = 15).

**Fig 11:**
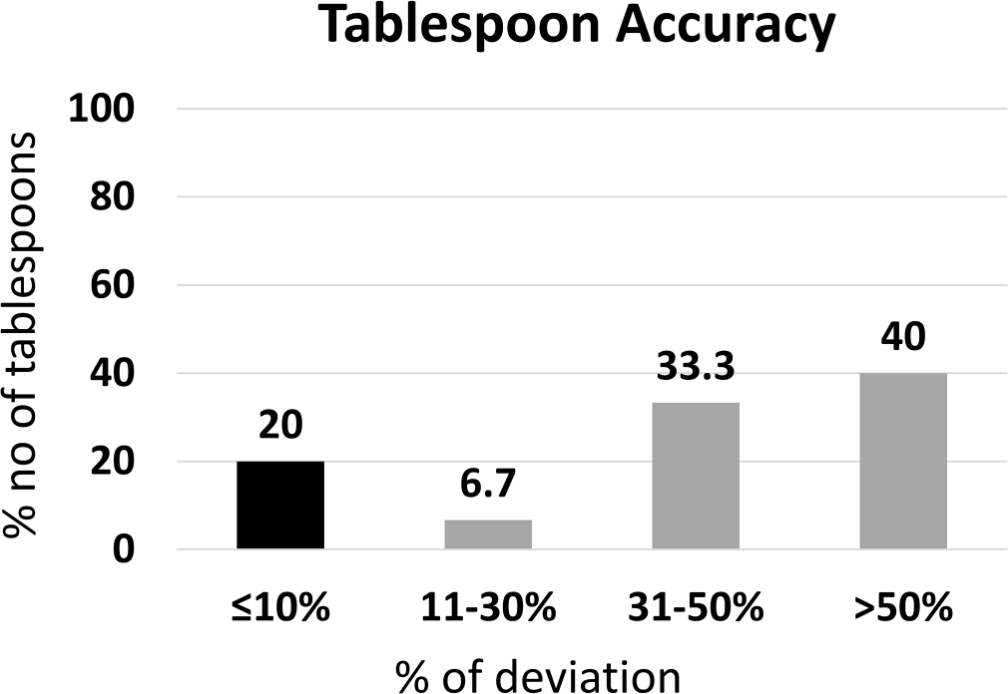
Deviation of household tablespoons from standard, 15 mL (n = 15).

From the 15 tablespoons, 20.0% contained ≤10% deviation from the expected 15.00 mL volume and complied with the USP criteria for measuring devices. Further, 6.7% had an 11–30% deviation, 33.3% had a 31–50% deviation, and 40.0% had a ≥ 50% deviation from the expected measuring volume (Fig 11).

These findings indicate substantial underperformance in volume accuracy among household tablespoons, with most failing to meet USP criteria.

#### Accuracy of teaspoons.

This subsection assessed 45 household teaspoons to evaluate their volume accuracy against the standard 5.00 mL measure.

The volume of teaspoons ranged between 2.89 mL to 7.76 mL. From the 45 teaspoons that were used in the study, 93.3% were recorded as having a measuring volume less than the expected 5.00 mL volume, 4.44% were higher than the expected 5.00 mL volume, and one teaspoon (2.22%) measured exactly 5.00 mL. Teaspoons showed the overall mean ± SD measuring volume of 4.02 ± 0.97 mL.

**Fig 12:**
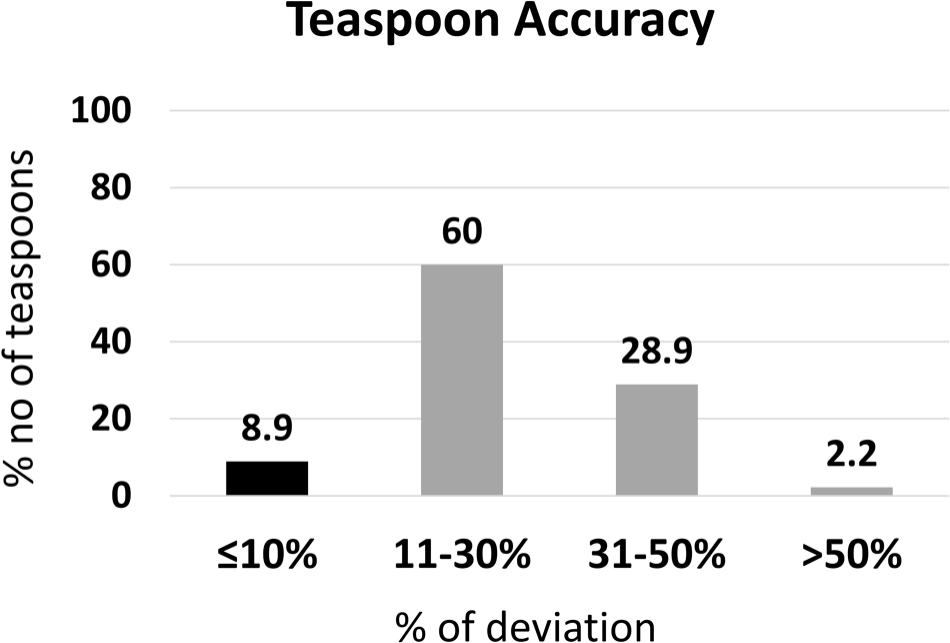
Deviation of household teaspoons from standard, 5 mL (n = 45).

From the 45 teaspoons, 8.89% had a ≤ 10% deviation of the expected 5.00 mL volume and complied with the USP criteria. Further, 60.0% of teaspoons had an 11–30% deviation from the expected measuring volume, while another 28.9% and 2.2% had 31–50% and ≥50% deviation, respectively (Fig 12).

The results highlight widespread inaccuracy among household teaspoons, with more than 90% failing to meet USP volume accuracy standards.

An independent samples t-test was conducted to assess the difference between the mean volumes measured by household spoons and their respective expected standard volumes. The results showed a significant difference for tablespoons, with measured volumes of 9.00 ± 3.39 mL compared to the expected 15.00 ± 0.00 mL (p ≤ 0.001). Similarly, teaspoons measured 4.02 ± 0.97 mL, which was significantly different from the expected volume of 5.00 ± 0.00 mL (p ≤ 0.001).

## Discussion

This study provides empirical confirmation of a largely overlooked issue, the continued use of inaccurate household dosing tools in medicine administration. Using a systematic experimental laboratory method within a cross-sectional design, this study evaluated the actual volumes delivered by dosing tools routinely used in a Sri Lankan community. The findings reveal widespread and systematic inaccuracies: over 93% of household spoons delivered volumes below expected standards, 62.5% of graduated measuring spoons failed USP specifications, and only 53.4% of measuring cups met accuracy standards across all tested volumes. These results demonstrate that measurement inaccuracy is not merely a user error problem but a fundamental device quality issue with significant implications for patient safety. The findings of this study urge that the choice of measuring aids and the inherent accuracy of dosing aids should not be undermined and call for regulatory attention and policy action to ensure the availability of accurate measuring devices with liquid medicines.

The results highlight considerable variability in accuracy across device types and volume levels. The accuracy of the minimum volume marks on measuring devices was found to be low. Only 53.4% of measuring cups passed all the volumes measured, and 86.2% passed the 5.00 mL measurement. Most measuring cups (minimum volume, (n) = 63.8%, 5 mL volume, (n) = 86.2%, and maximum volume, (n) = 94.8%) deviated within less than 10% and passed the USP criteria. In contrast, most (62.5%) of the measuring spoons failed to meet USP specifications, reinforcing the concern that even devices marketed as ‘graduated’ may fall short of acceptable standards. The study considered 5.00 mL as the standard volume of a teaspoon and 15.00 mL as the standard volume of a tablespoon. However, volumes measured by teaspoons ranged from 2.893 mL – 7.759 mL and volumes measured by tablespoons ranged from 4.252 mL – 15.043 mL. Volumes measured by most tablespoons (93.3%) and teaspoons (93.3%) were below standard, suggesting a systemic tendency toward underdosing when household tools are used. These findings point to a widespread risk of underdosing or overdosing, particularly when household utensils are used.

Findings demonstrate substantial variability in household teaspoon and tablespoon volumes, with measured capacities ranging from 2.893-7.759 mL and 4.252-15.043 mL respectively, far exceeding acceptable deviation limits. This variability is particularly concerning given that prescription habits in Sri Lanka continue to reference teaspoons or tablespoons as dosing units, despite long-standing recommendations for metric-based prescribing. The most widely used household spoons are the 5 mL teaspoon and 15 mL tablespoon, all of which have a wide range of designs and capacities [13].

Findings align with and extend previous research on household spoon inaccuracy. A study conducted in Greece reported the teaspoons’ capacity ranging from 2.5 mL to 7.3 mL, and tablespoons’ capacity ranged from 6.7 mL to 13.4 mL, showing considerable variability [14]. This study found comparable variability (teaspoons: 2.893-7.759 mL; tablespoons: 4.252-15.043 mL), with the critical additional finding that over 93% of both spoon types delivered less than their expected standard volumes, indicating a systematic tendency toward underdosing rather than random variability. These studies emphasized that teaspoons and tablespoons are proven to be erroneous tools for measuring and delivering liquid drugs.

Researchers have demonstrated that parents and caregivers often have low measuring accuracy when using household spoons for liquid medications, frequently resulting in underdosing or overdosing. For instance, a study in the USA reported that participants received a lower dose (8.4%) when using medium-sized spoons and a higher dose (11.6%) when using larger spoons [14]. Research in Ghana also indicated significant inaccuracies in the volume capacities of household spoons, leading to potential dosing errors [13]. One other case is where approximately 39.4% of parents made errors in measuring doses. Among these parents, those who used teaspoons or tablespoons were twice as likely to measure incorrectly compared to those who used a graduated device [16]. This pattern echoes findings from previous research in both high- and low-income settings, emphasizing that uncalibrated tools consistently introduce risk into routine medicine administration.

In the Sri Lankan context, the persistence of household measure terminology occurs at two critical points: at prescription (where some prescribers continue to write doses as ‘teaspoons’ or ‘tablespoons’) and at dispensing (where pharmacists translate metric doses into household measures when labeling repacked medications). This dual-source problem requires intervention at both prescribing and dispensing levels to achieve meaningful safety improvements.

The importance of accurate dosing is underscored by the need for precise drug delivery in medical treatments, where even minor deviations can have significant impacts, particularly for medications with narrow therapeutic windows. Efforts to improve outpatient safety must address both user education and upstream changes, such as ensuring calibrated dosing tools are supplied at the point of dispensing [4]. Pediatricians have been recommended to write prescriptions for liquid drugs in milliliters since the 1970s [15,16]. The inclusion of graduated dosing aids with prescription oral medications can help patients and caregivers use liquid medications more safely. Multiple studies have recommended the use of syringes as the best measuring aid for liquid medications due to their higher accuracy and ease of use, which can help ensure proper dosing and improve health outcomes [13,17,18].

Another significant issue is the inaccuracy of dosage delivery devices provided with products. While prior studies have primarily examined human errors in liquid dosage measuring, it is crucial to recognize that faulty devices can lead to inaccurate doses, regardless of user familiarity. This issue is common in low- and middle-income countries, where inaccuracies in liquid dosing devices are frequently reported. Research indicates that dosing cups and spoons often result in dosing errors, deviating from the intended 5 mL volume [3,19–21]. Findings of the present study corroborate these international observations, demonstrating that even graduated measuring devices marketed for medication use may fail to meet accuracy standards.

The results reveal a multi-level system failure requiring coordinated intervention. Addressing these risks calls for a coordinated response across regulatory bodies, manufacturers, and healthcare providers, with clear standards for dosing tool calibration and inclusion in medicine packaging. In the Sri Lankan context, specific actions are needed: Regulatory level: Mandatory inclusion of calibrated measuring devices with all liquid medications, with enforcement mechanisms for both imported and locally manufactured products. Quality control standards should specify acceptable deviation limits (e.g., USP ≤ 10%) for all dosing devices. Dispensing level: Bulk dispensing practices must be reformed to ensure every dispensed liquid medication is accompanied by a validated measuring device. Pharmacy dispensing guidelines should explicitly prohibit prescribing or labeling in household measure units (teaspoons/tablespoons). Prescriber level: Medical and dental professionals should prescribe exclusively in milliliters, with educational initiatives to phase out household measure terminology from clinical practice. Patient level: Public health campaigns should educate caregivers about the risks of household spoons and the importance of using only calibrated devices supplied with medications. Integrating these recommendations into practice may significantly reduce the likelihood of preventable medication errors, especially in pediatric and primary care settings.

This study has several limitations that should be acknowledged. First, convenience sampling from a single suburban area limits the generalizability of findings to other regions of Sri Lanka with different socioeconomic profiles, healthcare access, or device availability patterns. However, this sampling approach was appropriate for a device validation study, as the measurement properties tested are intrinsic to the devices themselves rather than dependent on household characteristics. The devices tested represent those actually in use within the community, providing ecologically valid data on real-world dosing practices.

Second, detailed household demographic data (income, education level, household composition) were not systematically collected. While this prevents analysis of associations between household characteristics and device type or accuracy, it does not compromise the validity of the study’s core findings. This study was designed to assess device accuracy against standardized specifications rather than to examine population-level behavioral patterns. Future research examining relationships between household characteristics and device selection or usage patterns could provide valuable complementary insights for targeting public health interventions.

Third, the sample size of graduated measuring spoons was small (n = 8), as these devices were not widely used in the study area. To strengthen generalizability, more extensive testing of graduated spoons and other commercially available liquid dosing aids across different regions would be valuable. Future research should assess a broader range of devices, including specialized measuring tools, to identify the most accurate dosing aids for liquid medications and support evidence-based recommendations for clinical practice and regulatory policy.

## Conclusion

This study documents substantial inaccuracies in commonly used liquid medication dosing tools in Sri Lankan households, including household spoons, measuring cups, and commercially produced graduated spoons. These findings call for regulatory mechanisms to ensure the availability and use of standardized, validated dosing aids with all dispensed liquid medicines. Complementary education initiatives targeting prescribers, dispensers, and patients are necessary to shift dosing practices toward safer, metric-based standards. Embedding calibrated tools and metric instructions into routine dispensing practices could help reduce preventable dosing errors, especially in pediatric and community care. Further research is warranted to evaluate additional device types and support evidence-informed guidance on optimal tools for liquid medication delivery. Recognizing and addressing this often neglected safety gap is essential for improving medication practices and safeguarding patients in low-resource health systems.

## Supporting information

S1 Data Volumetric data used to assess the accuracy of liquid measuring devices. (XLSX)

S2 Data Weighing method data used to assess the accuracy of liquid measuring devices. (XLSX)
